# Cytophagic histiocytic panniculitis, hemophagocytic lymphohistiocytosis and undetermined autoimmune disorder: reconciling the puzzle

**DOI:** 10.1186/1824-7288-40-17

**Published:** 2014-02-13

**Authors:** Claudia Pasqualini, Mauro Jorini, Ines Carloni, Mirella Giangiacomi, Valentina Cetica, Maurizio Aricò,, Fernando Maria de Benedictis

**Affiliations:** 1 Department of Mother and Child Health, Azienda Ospedaliero-Universitaria, Ancona, Italy; 2 Department of Pathology, Azienda Ospedaliero-Universitaria, Ancona, Italy; 3 Department of Pediatric Hematology-Oncology, Meyer Children’s Hospital, Florence, Italy; 4 Istituto Toscano Tumori, (I.T.T.), Florence, Italy

**Keywords:** Panniculitis, Histiocytes, Hemophagocytosis

## Abstract

Cytophagic histiocytic panniculitis is a rare disease, associated with either nonmalignant conditions or subcutaneous panniculitis-like T-cell lymphoma, and often also associated with hemophagocytic lymphohistiocytosis (HLH). We report the case of a 11-year-old boy with a history of secondary HLH who, after a local trauma, developed a painful, indurated plaque over the right thigh associated with relapsing HLH. Histopathologic findings from skin biopsy specimens revealed significant lobular panniculitis with benign histiocytes showing hemophagocytosis. High-dose intravenous methylprednisolone and cyclosporine A treatment was highly effective. A genetic study after a new, relapsing episode of HLH revealed an heterozygous missense mutation on STX 11 gene inherited from the mother.

## Background

Cytophagic histiocytic panniculitis (CHP) is a rare disease, first described in 1980, characterized by infiltration of subcutaneous adipose tissue by benign-appearing T lymphocytes and phagocytic histiocytes (“bean bag cells”) [1]. CHP may be an isolated skin disease or associated with non-malignant conditions, such as infections, as well as malignancies, including subcutaneous panniculitis-like T-cell lymphoma (SPTL), a rare form of non-Hodgkin lymphoma infiltrating into subcutaneous adipose tissue [2]. Subcutaneous panniculitis has been reported in a small number of patients with hemophagocytic lymphohistiocytosis (HLH), a life threatening condition characterized by uncontrolled activation and proliferation of T-cells resulting in hypercytokinemia, proliferation of histiocytes and hemophagocytosis [3,4]. The familial form of HLH (FHL) is a genetically heterogeneous disorder caused by mutations in genes involved in the granule-dependent exocytosis pathway. Patients with FHL are unable to cope with common pathogens; thus upon infection with widely diffused agents as cytomegalovirus and Epstein Barr virus, but also less common as leishmania, they develop clinical symptoms and findings of HLH [5]. In order to facilitate the diagnosis of HLH, a set of diagnostic criteria have been developed by the Histiocyte Society [6].

About 40 cases of CHP have been reported so far, mostly in adults [2]. Patients with CHP may have three different clinical courses, mainly depending on isolated presentation or association with HLH. Some patients rapidly progress and often die within one year, due to sepsis, coagulation disorders and multi-organ failure. Others have recurrent bouts of reactivation and may survive for years. Other patients respond well to treatment and may have a normal life [7,8].

We report a severe HLH-associated CHP successfully treated with systemic corticosteroids and cyclosporin A in a child with relapsing HLH and persistent ANA positivity at follow-up.

## Case presentation

A previously healthy 11-year-old boy was admitted to our department with persistent fever and increasing dyspnea. The family history was silent. On admission, the patient had high-grade fever and moderate respiratory distress. Chest examination revealed dullness on percussion and absent breath sound on the right hemithorax. Laboratory tests showed pancytopenia, impaired liver function, raised triglycerides and ferritin levels and low albumin value, without coagulopathy. Anti-nuclear antibodies (ANA) were positive 1:128 with homogeneous pattern. Autoantibody profile (anti-endonuclear, -cardiolipin, -beta2 glycoprotein I, -thyroid, -neutrophil cytoplasm, -mitochondrial and -smooth muscle antibodies) was negative. Blood and urine culture were negative. Virological tests revealed prior/remote Epstein-Barr virus (EBV) infection and negative serology for hepatitis viruses A, B, C, or cytomegalovirus. Chest x-ray showed homogeneous opacity of the right hemithorax. Ultrasound of the chest and abdomen revealed massive pleural effusion, and pericholecystic, perisplenic and perihepatic fluid collection. A chest tube was positioned and a total of 270 ml of cloudy pleural fluid was drained: cytology and biochemical analysis revealed leukocytes 490/mcL (80% lymphocytes), glucose 93 mg/dL, proteins 4,3 gr/dL, lactate dehydrogenase 3948 IU/L; no bacteria were found at the Gram and acid fast bacilli stain; the culture was negative. Bone marrow aspirate showed an increased number of histiocytes phagocyting red cells and platelets. The number of T, B and NK cells, and granzyme B concentration were within normal ranges. No mutations in *PRF1*, *UNC13D*, *STXBP2,* the genes most frequently associated with familial HLH, were found. HLH secondary to undetermined autoimmune disorder was diagnosed, and treatment with oral dexamethasone (initially 10 mg/m2 for 2 weeks followed by 5 mg/m2 for 2 weeks, 2.5 mg/m2 for 2 weeks, 1.25 mg/m2 for one week, and one week of tapering), cyclosporin (6 mg/kg daily) and intravenous etoposide (150 mg/m2 twice weekly for 2 weeks and then weekly) was administered for 8 weeks. All symptoms gradually resolved and the patient was maintained on a regular follow-up.

**Figure 1:**
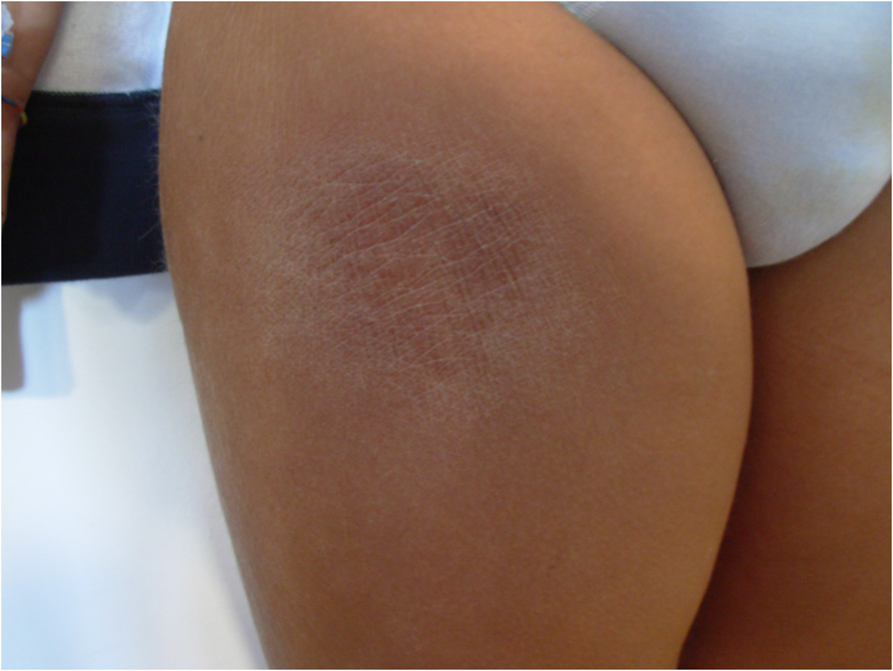
Hyperpigmented, indurated plaque with hyperkeratosic central area over the right thigh.

**Figure 2:**
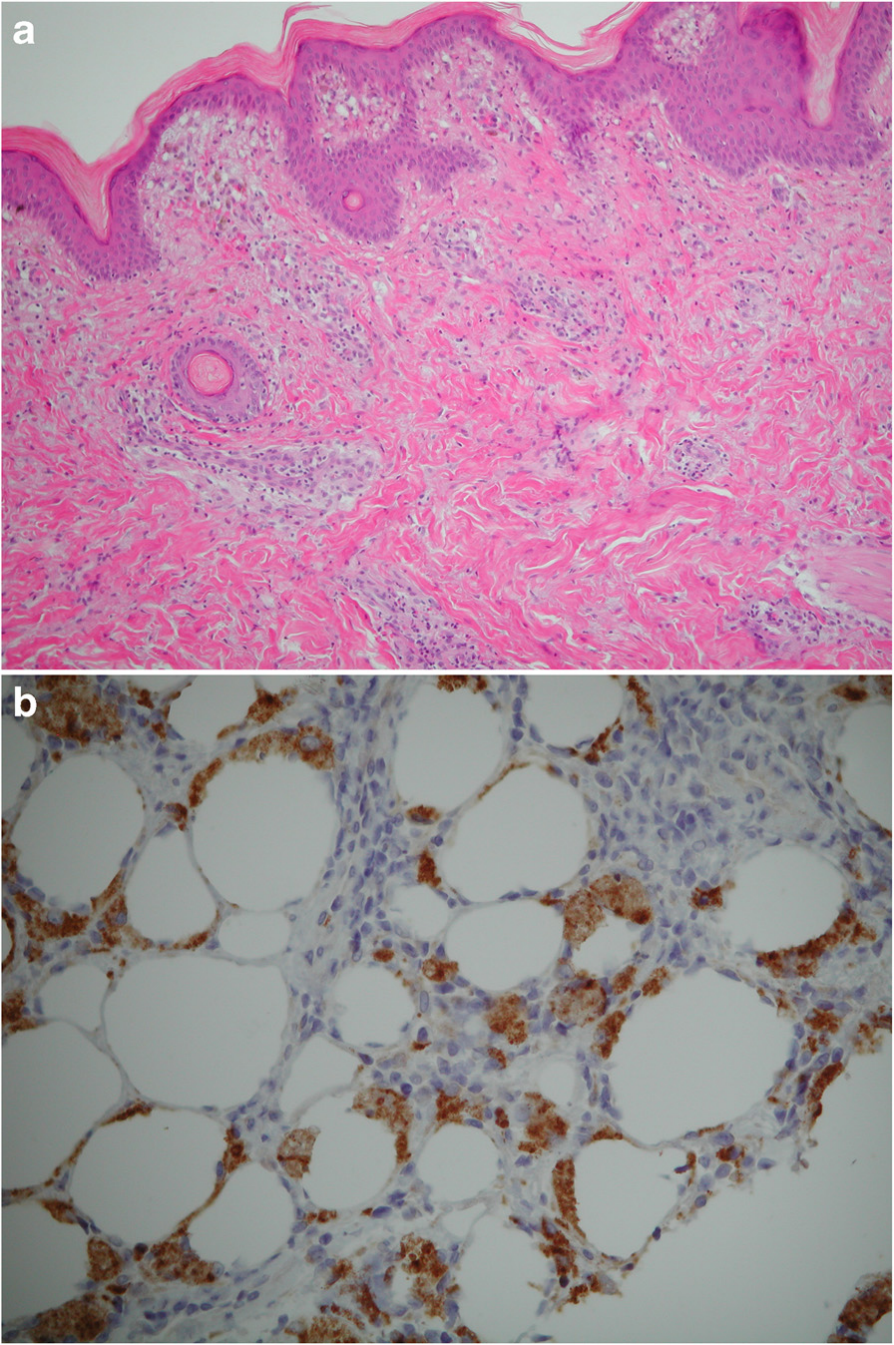
**Skin biopsy specimen. a)** Hematoxylin-eosin stain (4x) showing subcutaneous infiltrate, mainly constituted of mononuclear cells; **b)** Immunohistochemical stain (20x) showing infiltrating histiocytes expressing CD68.

One year later, the child presented with spiking fever and painful swelling of the right thigh which had gradually developed some days after a local trauma. Physical examination revealed a warm, painful, indurated plaque (10x12 cm diameter) over the right thigh. Liver and spleen were mildly enlarged. Routine laboratory tests were non-contributory. ANA were positive 1:1024 with granular pattern. Over a few days, the lesion progressively enlarged and a brownish, hyperpigmented, hyperkeratosic central area appeared (Figure 1). Laboratory tests and bone marrow aspirate were consistent with reactivation of HLH. Skin biopsy revealed a mixed septal and lobular inflammatory infiltrate of benign-appearing histiocytes, plasma cells and lymphocytes, and diffuse fat necrosis (Figure 2a); extensive hemophagocytosis by histiocytic cells was also evident. At the immuno-histochemical staining, nearly all the infiltrating lymphocytes expressed the phenotype of cytotoxic T-cell: CD2, CD3, CD5, CD8, but not CD4; CD7 expression was weak to negative; most histiocytes expressed CD68 (Figure 2b). Polymerase chain reaction analysis of the T-cell receptor-gamma chain gene rearrangement confirmed the absence of clonality. Testing for EBV virus by *in situ* hybridization was negative. A diagnosis of HLH-associated CHP was made. High-dose pulse of intravenous methylprednisolone (30 mg/kg/day for 3 days) was started, followed by a combination of dexamethasone (0.25 mg/kg daily) and cyclosporin A (6 mg/kg daily). A dramatic clinical improvement was observed. Dexamethasone was discontinued after 8 weeks, while cyclosporin A was maintained for 12 months.

Six months after discontinuation of cyclosporine A therapy, the patient was admitted for reactivation of HLH (Table 1). Thus, a more extensive genetic study for familial HLH was performed. Mutation analysis revealed heterozygous missense mutation (c.991G > A p.V267M) in the *STX11* gene. In silico analysis performed using the web query tools (Pmut, Polyphen, SIFT) confirmed that this mutation is not tolerated. Combined treatment with steroids (high-dose pulse of intravenous methylprednisolone 30 mg/kg/day for 3 days, followed by dexamethasone 0.10 mg/kg daily) and cyclosporine A (5 mg/kg daily) was reinstituted for 6 weeks and 12 months, respectively. Clinical and laboratory remission was sustained at 6 month follow-up. Over the years, ANA title remained persistently positive 1:256 to 1:1024 with granular pattern.

**Table 1:** Clinical features and laboratory findings at first admission and at relapses

	**1st admission**	**1st relapse**	**2nd relapse**
*Clinical features*	Spiking fever	Spiking fever	Fever
	Respiratory distress	Swelling of the right tight	Cough
	Absent breath sound on right hemithorax	Liver and spleen enlargement	
*Laboratory findings*			
Red blood cells (x10^6^)	4.250	4.140	4.790
White blood cells (x10^3^)	1.63	2.54	2.88
Platelets (x10^3^)	90	145	203
AST/ALT (U/L)	122/101	144/104	55/65
Triglycerides (mg/dL)	508	180	240
Ferritin (ng/ml)	7.547	670	249
Albumin (g/dL)	3,3	3,9	3,5
Antinuclear antibodies	1:128	1:1024	1:516
	Homogeneous pattern	Granular pattern	Granular pattern
Bone marrow	Phagocyting histiocytes	Phagocyting histiocytes	

## Conclusions

Our case is worthy of description for several reasons. First, there is no mention in the literature of autoimmune involvement in patients with CHP. ANA were positive at the presentation of HLH and their values remained persistently high over the years. The meaning of ANA positivity in the picture of systemic disease remains intriguing. Ravelli et al. [9] suggested that ANA positivity is the hallmark of a specific subset of patients with juvenile idiopathic arthritis which should be classified separately for their different prognosis [10]. Whether persistent ANApositivity in our patient may represent a different setting of the immune system cannot be defined at present. Furthermore, a local trauma was revealed some days before panniculitis was evident. To our knowledge, this finding has not been reported in CHP and trauma might act as precipitating factor.

The spectrum of mutations involved in FHL has expanded since the original report of perforin defect in 1999, with mutations identified in the *UNC13D* (FHL3), *STX11* (FHL4) and *STXBP2* (FHL5) genes [11]. While original reports of FHL4 were restricted to families of Turkish/Kurdish origin, patients of different origins have been recently identified with defect in *STX11*[11,12]. The relevance of cooperation between the syntaxins and other proteins involved in the degranulation machinery is being progressively elucidated [13]. Recent studies in patients with FHL suggested that monoallelic mutation in FHL-related genes may behave as predisposing factor for several human disorders [14-16]. The working hypothesis is that partial impairment of the cellular cytotoxicity machinery may predispose, or contribute, to several disorders in which the immune system plays a significant role [11,12]. Our patient bears a monoallelic, novel STX11 mutation, which was predicted to be pathogenic by in silico analysis.

Once CHP is suspected, the diagnosis relies mainly on histopathology findings. In such context, discriminating between CHP and SPTL is therapeutically important because nonmalignant CHP often improves under pulses of high-dose intravenous methylprednisolone and cyclosporine A [17], whereas most cases of SPTL may be best treated with more aggressive therapy. Marzano et al. [18] suggested that these conditions might span a clinical-pathological spectrum in which there is a natural progression from CHP to SPTL. Since the distinction of CHP from SPTL is difficult and CHP might be a precursor of SPTL [19], some authors proposed to use the term “panniculitis-like subcutaneous lymphoma with cytophagocytosis” instead of CHP, even when T-cell clonality was not documented [20]. This approach would have a beneficial effect on treatment planning towards oncological rather than anti-inflammatory therapy. Bader-Meunier et al. [21] recently emphasized that HLH-associated CHP may be diagnosed despite monoclonal T-cell proliferation that mimics SPTL and is best treated by prednisone and cyclosporine A, at least in children. It has been suggested that this florid clonal T-cell proliferation is reactive, probably driven by a strong immune reaction against EBV infection [22]. Furthermore, Huppmann et al. [23] confirmed that molecular studies are diagnostically helpful but not specific, since the absence of clonality does not rule out the diagnosis of SPTL.

The diagnosis of CHP is challenging, but it was supported by several data in our case. Although many histopathology findings are common to both CHP and SPTL, rimming of the fat vacuoles by atypical lymphoid cells is a useful diagnostic feature for SPTL [24]. Furthermore, the development of HLH is extremely rare, has an aggressive course and is typically associated with a poor outcome in children with SPTL [25]. In our patient, the rimming pattern was absent in the skin infiltrate and the outcome of HLH was favourable.

Another entity within the differential diagnosis spectrum of lobular panniculitis is lupus erythematosus panniculitis (LEP) [26], a rare variant of lupus erythematosus which can present as an isolated phenomenon and is most commonly localized to upper arm. The presence of HLH in LEP, has been rarely described [27]. Male gender, involvement of the thigh, good response to therapy, lack of relapse after treatment discontinuation, and absence of characteristic histopathology findings of lupus erythematosus supported the diagnosis of CHP.

The remission of the symptoms of CHP and HLH under the association of high-dose intravenous methylprednisolone and cyclosporine A in our patient supports that this combination is the treatment of choice for nonmalignant CHP associated with HLH. Cytotoxic chemotherapy should be considered for relapsing/refractory disease or more severe forms [28]. High-dose chemotherapy followed by autologous peripheral blood stem cell transplantation may be necessary for the treatment of particularly aggressive CHP [29].

The small number of reported patients with HLH-associated CHP does not permit to hypothesize any factor that may predict the clinical course or guide the optimal treatment. Searching for biological features such as underlying genetic mutations may help to better understand the variability of the clinical course and the response to treatment. Prompt diagnosis of CHP and HLH, close cooperation between rheumatologists, hematologists, dermatologists and pathologists, and continued follow up remain an imperative matter that can make the difference between life and death.

## Consent

Written informed consent was obtained from the parents of the patient for publication of this Case Report and any accompanying images. A copy of written consent is available for review by the editor-in-chief of this journal.

## Abbreviations

CHP: Cytophagic histiocytic panniculitis; SPTL: Subcutaneous panniculitis-like T-cell lymphoma; HLH: Hemophagocytic lymphohistiocytosis; LEP: Lupus erythematosus panniculitis.

## Competing interests

The authors declare that they have no competing interests.

## Authors’ contributions

CP identified the case, helped in making the diagnosis and worked on bibliography. MJ was involved in the diagnostic process, therapeutic decisions and follow up. IC drafted the manuscript. MG made substantial contribution to analysis and interpretation of immunohistochemical data. VC performed mutation analysis. MA supervised mutation analysis and revised the manuscript. FMdB supervised the diagnostic and therapeutic approach, and critically revised the manuscript. All authors read and approved the final manuscript.

## References

[B1] Winkelmann RK Bowie EJ Haemorrhagic diathesis associated with benign histiocytic, cytophagic panniculitis and systemic histiocytosis Arch Int Med 1980 140 1460 1463 10.1001/archinte.1980.00330220038015 7436642

[B2] Aronson IK Worobec SM Cytophagic histiocytic panniculitis and hemophagocytic lymphohistiocytosis: an overview Dermatol Ther 2010 23 389 402 10.1111/j.1529-8019.2010.01339.x 20666826

[B3] Gupta S Weitzman S Primary and secondary hemophagocytic lymphohistiocytosis: clinical features, pathogenesis and therapy Expert Rev Clin Immunol 2010 6 137 154 10.1586/eci.09.58 20383897

[B4] Aricò M Janka G Fischer A Henter JI Blanche S Elinder G Martinetti M Rusca MP Hemophagocytic lymphohistiocytosis. Report of 122 children from the International Registry. FHL Study Group of the Histiocyte Society Leukemia 1996 10 197 203 8637226

[B5] Ansuini V Rigante D Esposito S Debate around infection-dependent hemophagocytic syndrome in paediatrics BMC Infect Dis 2013 13 15 doi:10.1186/1471-2334-13-15 10.1186/1471-2334-13-15 23324497 PMC3549728

[B6] Henter JI Horne A Aricó M Egeler RM Filipovich AH Imashuku S Ladisch S McClain K Webb D Winiarski J Janka G HLH-2004: Diagnostic and therapeutic guidelines for hemophagocytic lymphohistiocytosis Pediatr Blood Cancer 2007 48 124 131 10.1002/pbc.21039 16937360

[B7] Aronson IK West DP Variakojis D Malkinson FD Wilson HD Zeitz HJ Fatal panniculitis J Amer Acad Dermatol 1985 12 535 551 10.1016/S0190-9622(85)70076-X 3989012

[B8] White JW Winkelmann RK Cytophagic histiocytic panniculitis is not always fatal J Cutan Pathol 1989 16 137 144 10.1111/j.1600-0560.1989.tb00028.x 2768594

[B9] Ravelli A Felici E Magni-Manzoni S Pistorio A Novarini C Bozzola E Viola S Martini A Patients with antinuclear antibody-positive juvenile idiopathic arthritis constitute a homogeneous subgroup irrespective of the course of joint disease Arthritis Rheum 2005 52 826 832 10.1002/art.20945 15751057

[B10] Ravelli A Varnier GC Oliveira S Castell E Arguedas O Magnani A Pistorio A Ruperto N Magni-Manzoni S Galasso R Lattanzi B Dalprà S Battagliese A Verazza S Allegra M Martini A Antinuclear antibody-positive patients should be grouped as a separate category in the classification of juvenile idiopathic arthritis Arthritis Rheum 2011 63 267 275 10.1002/art.30076 20936630

[B11] Cetica V Pende D Griffiths GM Aricò M Molecular basis of familial hemophagocytic lymphohistiocytosis Haematologica 2010 95 538 541 10.3324/haematol.2009.019562 20378576 PMC2857182

[B12] Sieni E Cetica V Mastrodicasa E Pende D Moretta L Griffiths G Aricò M Familial hemophagocytic lymphohistiocytosis: a model for understanding the human machinery of cellular cytotoxicity Cell Mol Life Sci 2012 69 29 40 10.1007/s00018-011-0835-y 21990010 PMC11114696

[B13] Hackmann Y Graham SC Ehl S Höning S Lehmberg K Aricò M Owen DJ Griffiths GM Syntaxin binding mechanism and disease-causing mutations in Munc18-2 Proc Natl Acad Sci U S A 2013 110 E4482 E4491 doi:10.1073/pnas.1313474110. Epub 2013 Nov 5 10.1073/pnas.1313474110 24194549 PMC3839780

[B14] Aricò M Boggio E Cetica V Melensi M Orilieri E Clemente N Cappellano G Buttini S Soluri MF Comi C Dufour C Pende D Dianzani I Ellis SR Pagliano S Marcenaro S Ramenghi U Chiocchetti A Dianzani U Variations of the UNC13D gene in patients with autoimmune lymphoproliferative syndrome PLoS One 2013 8 e68045 10.1371/journal.pone.0068045 23840885 PMC3698121

[B15] Cappellano G Orilieri E Comi C Chiocchetti A Bocca S Boggio E Bernardone IS Cometa A Clementi R Barizzone N D'Alfonso S Corrado L Galimberti D Scarpini E Guerini FR Caputo D Paolicelli D Trojano M Figà-Talamanca L Salvetti M Perla F Leone M Monaco F Dianzani U Variations of the perforin gene in patients with multiple sclerosis Genes Immun 2008 9 438 444 10.1038/gene.2008.35 18496551

[B16] Ciambotti B Mussolin L D'Amore ES Pillon M Sieni E Coniglio ML Ros MD Cetica V Aricò M Rosolen A Monoallelic mutations of the perforin gene may represent a predisposing factor to childhood anaplastic large cell lymphoma J Pediatr Hematol Oncol 2013 Dec 4. [Epub ahead of print]. doi:10.1097/MPH.0000000000000073 10.1097/MPH.0000000000000073 24309606

[B17] Nakane S Kawabe Y Eguchi K Kita A Mizokami A Yamasaki H NAgataki S A case of cytophagic histiocytic panniculitis: successful treatment of recurrent attacks with steroid pulse therapy and oral cyclosporine A Clin Rheumatol 1997 16 417 421 10.1007/BF02242462 9259259

[B18] Marzano AV Berti E Paulli M Caputo R Cytophagic histiocytic panniculitis and subcutaneous panniculitis-like T-cell lymphoma: report of 7 cases Arch Dermatol 2000 136 889 896 10890991 10.1001/archderm.136.7.889

[B19] Willemze R Jansen PM Cerroni L Berti E Santucci M Assaf C Canninga-van Dijk MR Carlotti A Geerts ML Hahtola S Hummel M Jeskanen L Kempf W Massone C Ortiz-Romero PL Paulli M Petrella T Ranki A Peralto JL Robson A Senff NJ Vermeer MH Wechsler J Whittaker S Meijer CJ EORTC Cutaneous Lymphoma Group EORTC Cutaneous Lymphoma Group. Subcutaneous panniculitis-like T-cell lymphoma: definition, classification, and prognostic factors: an EORTC Cutaneous Lymphoma Group Study of 83 cases Blood 2008 111 838 845 10.1182/blood-2007-04-087288 17934071

[B20] Wick MR Patterson JW Cytophagic histiocytic panniculitis - a critical reappraisal Arch Dermatol 2000 136 922 924 10890995 10.1001/archderm.136.7.922

[B21] Bader-Meunier B Fraitag S Janssen C Brochard K Lamant L Wouters C Bodemer C Clonal cytophagic histiocytic panniculitis in children may be cured by Cyclosporine A Pediatrics 2013 132 e545 10.1542/peds.2012-3256 23858422

[B22] Ahn JS Rew SY Shin MG Kim HR Yang DH Cho D Kim SH Bae SY Lee SR Kim YK Kim HJ Lee JJ Clinical significance of clonality and Epstein-Barr virus infection in adult patients with hemophagocytic lymphohistiocytosis Am J Hematol 2010 85 719 722 10.1002/ajh.21795 20652965

[B23] Huppmann AR Raffeld M Pittaluga S Jaffe ES Subcutaneous panniculitis-like T-cell lymphoma in the pediatric age group: a lymphoma of low malignant potential Pediatr Blood Cancer 2013 60 1165 1170 10.1002/pbc.24462 23382035 PMC6324177

[B24] Koh MJ-A Sadarangani SP Chan YC Chan MY Tan AM Tan SH Tay YK Ng SB Aggressive subcutaneous panniculitis-like T-cell lymphoma with hemophagocytosis in two children (subcutaneous panniculitis-like T-cell lymphoma) J Am Acad Dermatol 2009 61 875 881 10.1016/j.jaad.2009.01.045 19744747

[B25] Merritt BY Curry JL Duvic M Vega F Sheehan AM Curry CV Pediatric subcutaneous panniculitis-like T-cell lymphoma with features of hemophagocytic syndrome Pediatr Blood Cancer 2013 60 1916 1917 10.1002/pbc.24638 23868752 PMC4094035

[B26] Park HS Choi JW Kim BK Cho KH Lupus erythematosus panniculitis: clinicopathological, immunophenotypic, and molecular studies Am J Dermatopathol 2010 32 24 30 10.1097/DAD.0b013e3181b4a5ec 20098081

[B27] Lonceint J Sassolas B Lefur JM Guillet G Leroy JP Panniculitis and macrophage activating syndrome in a child with lupus erythematosus Ann Dermatol Venereol 2001 128 1339 1342 11908139

[B28] Ito M Ohira H Miyata M Suzuki T Sato Y Kaise S Nishimaki T Sakuma H Nihei Y Iwatsuki R Cytophagic histiocytic panniculitis improved by combined CHOP and cyclosporin A treatment Intern Med 1999 38 296 301 10.2169/internalmedicine.38.296 10337947

[B29] Koizumi K Sawada K Nishio M Katagiri E Fukae J Fukada Y Tarumi T Notoya T Abe R Kobayashi H Koike T Effective high-dose chemotherapy followed by autologous peripheral blood stem cell transplantation in a patient with the aggressive form of cytophagic histiocytic panniculitis Bone Marrow Transplant 1997 20 171 173 10.1038/sj.bmt.1700858 9244423

